# Activity of crude extract of the sexual renal segment from C*rotalus durissus *on spermatic kinematics of thawed dog semen

**DOI:** 10.1186/1753-6561-8-S4-P150

**Published:** 2014-10-01

**Authors:** Cristiane Scavuzzi Moura, Chirlane Castro Silva, Sildivane Valcácia Silva, Ellen Cordeiro, Bento Silva, Ranilson S Bezerra, Maria Madalena Pessoa Guerr

**Affiliations:** 1 Laboratório de Doenças Nutricionais e Metabólicas de Ruminantes - Universidade Federal Rural de Pernambuco, Recife, PE, 52171-900, Brazil; 2 Laboratório de Enzimologia, Universidade Federal de Pernambuco, Recife, PE, 50670-901, Brazil; 3 Centro de Biotecnologia, Universidade Federal da Paraíba, João Pessoa, PB, 58051-900, Brazil; 4 Laboratório de Andrologia, Universidade Federal Rural de Pernambuco, Recife, PE, 52171-900, Brazil; 5 Departamento de Medicina Veterinária, Universidade Federal Rural de Pernambuco, Recife, Brasil

## Background

Males of the order *Squamata *have similar structure to the seminal vesicles of mammals called Renal Sexual Segment (RSS). The RSS regions are hypertrophied kidney ducts, androgen-dependent, responsible for providing secretions (complex of glycogen, mucopolysaccharides, mucoproteins and lipids) that nourishes and keeps the sperm during copulation [1,3], acting accessorily on reproductive cycle, once when mixed with sperm, and nutrient function, also act as activating agent of sperm transferred to the cloaca in the female during intercourse [2,3]. Valverde (personal communication, April 6, 2010) tested the crude extract of the renal sexual segment *Crotalus durissus *in bovine semen and the results showed a significant increase in sperm motility and vigor of the samples, but this activity was lost over time storage of the extract. The promising data in this study aimed to evaluate the effect of the addition of crude extract of the RSS from *C. durissus *on spermatic kinematics of thawed dog semen.

## Methods

Dog semen samples (n = 3) cryopreserved in tris-yolk-glycerol (7%) were thawed (37ºC/30s) and divided into three groups: G1=semen+48µg/mL extract unheated, G2=semen+48µg/mL heated extract (60ºC/30min) and G3=semen. The extract used was diluted in Tris buffer (0.25M Tris, 0.09M citric acid, fructose 0.06M, pH 6.8) to 0.2 mg/mL. After treatment, semen samples were incubate at room temperature (28°C) and analyse to Kinematics by Computer Assisted Semen Analysis method (CASA) immediately after the extract addition (T0) and 1h (T1) and 4 h (T4) of incubation. Data were analyzed by ANOVA and Tukey-Kramer test with 5% significance level. Results were expressed as Mean ± SD for G1, G2 and G3, respectively.

## Results and conclusions

In T0, the total motility (TM) of the sperm showed 54,60 ± 11,36; 46,27 ± 18,68; 47,47 ± 17,14, while for the progressive motility (PM) was 20,10 ± 4,25; 19,50 ± 3,70; 18,07 ± 4,24 to G1, G2 e G3. During T1, the total motility (TM) of the sperm showed 38,33 ± 12,06; 41,53 ± 6,82; 32,00 ± 10,32, while for the progressive motility (PM) was 11,30 ± 4,47; 10,87 ± 3,25; 9,00 ± 5,96 to G1, G2 e G3. In T4, the total motility (TM) of the sperm showed 41,27 ± 5,48; 32,47 ± 8,28; 22,77 ± 14,26, while for the progressive motility (PM) was 12,43 ± 6,81; 8,63 ± 5,59; 8,70 ± 7,41 to G1, G2 e G3, respectively. Although there was a tendency to higher levels of both total motility, progressive as after 1h and 4h incubation in Groups G1 and G2 in the control group, No significant difference (P > 0.05) was observed among experimental groups at each time of analysis to sperm total motility. Thus, we conclude that the addition of crude extract of the sexual renal segment from *Crotalus durissus *has no influence on spermatic kinematics of the thawed dog semen.
